# Relationship between polymorphism of insulin receptor gene, and adiponectin gene with PCOS

**Published:** 2013-03

**Authors:** Fahimeh Ramezani Tehrani, Maryam Daneshpour, Somayeh Hashemi, Maryam Zarkesh, Feridoun Azizi

**Affiliations:** 1 *Reproductive Endocrinology Research Center, Research Institute for Endocrine Sciences, Shahid Beheshti University of Medical Sciences, Tehran, Iran.*; 2 *Obesity Research Center, Research Institute for Endocrine Sciences, Shahid Beheshti University of Medical Sciences, Tehran, Iran.*; 3 *Endocrine Research Center, Research Institute for Endocrine Sciences, Shahid Beheshti University of Medical Sciences, Tehran, Iran.*

**Keywords:** *Receptor*, *Insulin/ genetics*, *Adiponectin/ genetics*, *Polycystic ovary syndrome*, *Polymorphism*, *Genetic/ genetics*

## Abstract

**Background: **Polycystic ovary syndrome (PCOS) is a complex disease having both genetic and environmental components and candidate genes on obesity and insulin metabolism have been hypothesized to be involved in its etiology.

**Objective:** We examined the possible association of adiponectin and insulin receptor gene polymorphisms with PCOS.

**Materials and Methods: **A total of 186 women with PCOS using NIH criteria and 156 healthy women were recruited. Their samples were genotyped for the polymorphism in exon 17 and 8 of the insulin receptor gene or exon and intron 2 of the adiponectin gene.

**Results:** The distributions of genotypes and alleles of both polymorphisms were not different in women with PCOS and controls. There was no significant differences on the anthropometric and hormonal profiles of various adiponectin and insulin receptor genes polymorphisms among both groups.

**Conclusion:** Adiponectin and insulin receptor gene polymorphisms are not associated with PCOS in a sample of Iranian population.

## Introduction

Polycystic ovary syndrome (PCOS), the most common gynecological endocrinopathy, is characterized by chronic anovulation and hyperandrogenism (1, 2). The National Institute of Child Health and Human Development Conference of PCOS (NIH) definded PCOS as existance of hyper - androgenism and /or hyperandrogenemia, oligoovulation, ultrasound pattern of polycystic ovaries and the exclusion of other known disorders (3). 

However the exact etiology of PCOS is unclear, androgen excess is suggested to play a key role. Androgen levels primarily increase by the ovaries with a smaller contribution from the adrenals and peripheral adipose tissue. High levels of androgen interfere with hypothalamic sensitivity to negative feedback from the ovary, thereby increasing GnRH pulse frequency (4). This persistently rapid pulse frequency favors increased LH secretion, which consequently stimulates the ovarian theca cells to produce more androgens (5). The relative decrease in FSH secretion leads to less aromatization of androgens to estradiol. Studies on homozygote twins or sisters suggested a genetic origin for this disorder, but the genetic components of this disorder have not been entirely illustrated (6-11). 

Over the past decade, a number of candidate genes involved in steroidogenesis, the insulin-signaling pathway, gonadotropin secretion and chronic inflammation have been investigated to ascertain the gene possibly involved in susceptibility to PCOS, but results have been contraversial (12, 13). Insulin resistance and central obesity are considered the two most common features of PCOS (14). 

Although several studies have been conducted to identify the associations of PCOS with various candidate genes involved in obesity and insulin resistance, no single common defect has yet been documented (15-17). The role of obesity in the pathophysiology of PCOS can be partly explained by the lower serum concentration of adiponectin in PCOS women (18). There are studies documenting positive results on the association between adiponectin gene polymorphism and the risk of PCOS (18-20), but this association was not observed in study on Greek women (14, 18, 20). 

Ranjzad *et al* in a study in Iran observed that “there is no significant difference in genotype and allele frequencies between the women with PCOS and controls for the rs2059806, rs1799817, rs1501299, rs6256, and rs757343 polymorphisms either before or after adjustment for confounding factors including age and BMI. However, the ADIPOQ rs2241766 ‘‘TT’’ genotype compared with ‘‘TG and GG’’ genotypes was associated with a 1.93-fold increased risk for PCOS”(19). 

Regarding ethnic differences and lack of consensus on genetic i involving in PCOS (21), we decided to conduct a study in Iranian population to compare the frequency of insulin receptor (INSR) (exon 17 and 8) and adiponectin (ADIPOQ) (exon and intron 2) genes polymorphisms between Iranian women with PCOS and normal controls. Two SNPs for each of these genes were selected based on their commonly use in previous genetic epidemiology studies and high degree of heterozygosity (13, 19).

## Materials and methods

The present cross-sectional study was carried out on non-menopause women, aged 20-40 years, presented in Reproductive Endocrine Research Center in 2011. Two groups were selected from among these women. 

A group of 186 women diagnosed with PCOS based on NIH diagnostic criteria for PCOS were recruited from the endocrinology clinic as a case group and a control group of 156 healthy non hirsute normo-ovulatory women were enrolled. Women with other endocrine disorders or a history of pelvic surgery were excluded. 

The study protocol was approved by the Ethics Committee of the Research Center for Endocrine Disorders, and all women studied gave informed consent. For each subject a standard questionnaire requiring data on their age, age at menarche, reproductive history, and family history of hirsutism, oligo/menorrhea and infertility was completed. The study participants underwent basic physical examinations that included measurement of height, weight and waist, hip and wrist circumferences, blood pressure, and hirsutism and acne scores and their blood samples were collected between the 3^rd^ and 6^th^ day of their spontaneous or progesterone induced menstrual cycles. 

PCOS was defined using the NIH criteria as the presence of anovulation and hyperandrogenism after exclusion of other known related disorders such as hyperprolactenemia, thyroid disorders, and non-classical adrenal hyperplasia. Anovulation was considered as history of eight or fewer menstrual cycles per year or a menstrual cycle length <21 or >40 days. 

Hyperandrogenism was considered as the presence of hirsutism modified Ferriman-Gallwey (mFG) score ≥8 and/or free androgen index (FAI), A4, and/ or Dehydroepiandrosterone sulfate (DHEAS) level above the upper 95th percentile of 40 healthy, non-hirsute eumenorrheic women. Insulin resistance was assessed using the homeostasis model (HOMA-IR) according to the following equation: Fasting blood glucose (FBG nmol/l) x fasting Insulin (mIU/l) /22.5. The FAI was calculated using the formula: [TT (nmol/L)×100/SHBG (nmol/L)] (22).

Androgen excess severity score was calculated according to the sum of scores of hyperandrogenemia and hirsutism. Hirsutism scores were categorized in to three groups (1=mild; FG≤12, 2=moderate; FG=13-20, 3=severe; FG>20), 2 score was added to categorized hirsutism score for each of those with high FAI, A4, and/or DHEAS. 

Anovulation severity was identified according to these three anovulation sub groups: 1=mild; menstrual cycle length less than 21 or between 40-60 days, 2=moderate; menstrual cycle length between 61-180 days, 3=severe; amenorrhea or menstrual cycle length >180days. PCOS severity score was calculated as sum of androgen excess score and anovulation score (min 2, max 11).

**Measurements**

Dehydroepiandrosterone sulfate (DHEAS), 17-hydroxyprogesterone (17OH-P), total testosterone (TT) and androstendion (A4) were measured by the enzyme immunoassay (EIA), (Diagnostic biochem Canada Co. Ontario, Canada). 

Sex hormone binding globulin (SHBG) was measured by the immunoenzymometric assay (IEMA), (Mercodia, Uppsala, Sweden). All ELISA tests were performed using Sunrise ELISA reader (Tecan Co. Salzburg, Austria). Luteinizing hormone (LH), follicle stimulating hormone (FSH), prolactin (PRL), and thyroid stimulating hormone (TSH) were measured by the immunoradimetric assay (IRMA), (Izotop, Budapest, Hungary) using gamma counter (Wallac Wizard, Turku, Finland). 

**Genotyping**

Genomic DNA was isolated from peripheral blood leukocytes of women with PCOS and controls using the salting out/proteinase K method (23). A total of four single nucleotide polymorphisms (SNPs) were genotyped using polymerase chain reaction-restriction fragments length polymorphism (PCR-RFLP), followed by 2% agarose gel electrophoresis (Table I). The digested products were analyzed by electrophoresis using a 2-3% agarose gel and visualized by ethidium bromide staining.

**Statistical analysis**

Continuous variables were checked for normality using the one-sample Kolmogorov-Smirnoff test. They are expressed as mean±standard deviation and/ or median and interquartile ranges, as appropriate. The categorical variables are expressed as percentages. The Chi-square test was used to confirm that the genotype data did not deviate from the Hardy-Weinberg equilibrium and to test for differences in allele frequency between the PCOS and the control groups. 

Distributions between groups are compared using the Kruskal-Wallis test followed with Mann-Whitney test and one-way ANOVA analysis, with Bonferroni correction, as appropriate. Data analysis was performed using the SPSS 15.0 PC package (SPSS Inc., Chicago, IL).

## Results

One hundred and eighty-six women with PCOS and 156 normo-ovulatory controls participated in this study. The mean and standard deviation of age among women in PCOS cases and controls were 26.6±5.6 and 30.8±5.6 years, respectively. The mean age of the control group was slightly older than that of the PCOS women, but this is not relevant when comparing genotype frequencies, as done in this study. 

Basic, reproductive and metabolic characteristics of study subjects are presented in table II. The prevalence of primary infertility and familial history of hirsutism among PCOS participants were significantly more than that among the normo-ovulatory ones (Table II). Genotype data was tested for deviation from Hardy-Weinberg equilibrium both separately (as case and control groups) and combined, and all SNPs were in Hardy-Weinberg equilibrium. The genotype, allele, and haplotype frequencies of SmaI, and BsmI polymorphisms of the adiponectin gene and PmlI, and NsiI polymorphisms of the INSR gene are shown in table III. Since there are differences in age and BMI of case and control groups, we have done analyses adjusting age and BMI, however results were similar.

**Figure 1:**
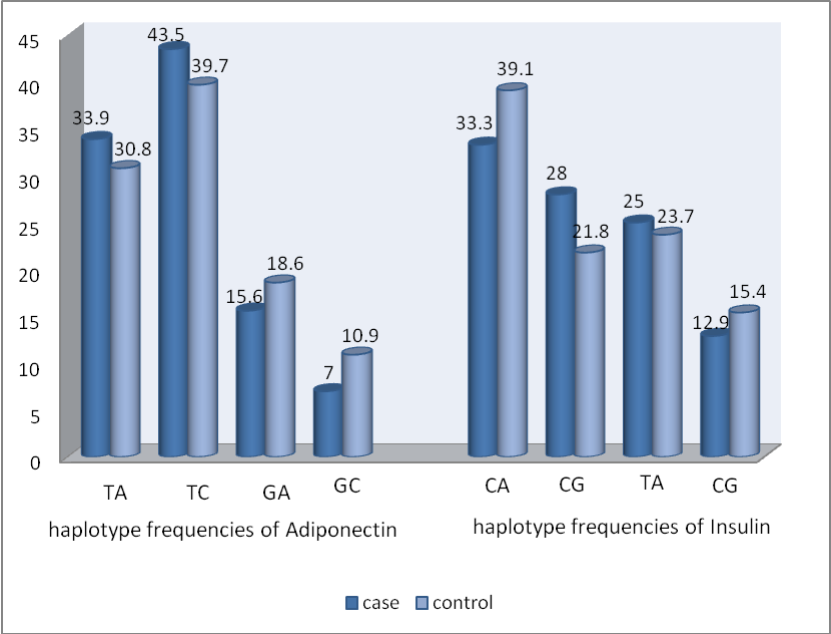
Haplotype frequencies (%) of studied SNPs in the case and control groups.

There were no differences in allele frequencies between case and control groups for the SNPs we studied in the adiponectin and INSR genes. The TT genotype, the T allele, and the TC haplotype of the SmaI polymorphism of the adiponectin gene presented the highest frequency in the case and control groups, [(76.3%, 77.4%, 43.5%) and (67.9%, 70.5%, 39.7)] respectively. The TT genotype, the T allele, and the CA haplotype of the PmlI polymorphism of the INSR gene presented the highest frequency in the case and control groups, [(61.3%, 61.3%, 33.3%) and (60.9%, 60.9%, 39.1)] respectively (Table III, Figure 1). 

The anthropometric, metabolic and hormonal profiles of the two groups (PCOS and controls) were not significantly different among various adiponectin and INSR genes polymorphisms. The presence of the TC haplotype of the adiponectin gene in controls had a significant association with higher waist circumference (Table IV).

**Table I:** Data for the studied markers in the Insulin Receptor (INSR), and Adiponectin (ADIPOQ) genes

Gene/SNP* **(SNP ID)**	**rs number**	**Location** **(Base change)**	**Reverse primer Forward primer**	**PCR program** **(35 cycles)**	**PCR fragment size (bp)**	**Restriction enzyme, Incubation temperature**	**Alleles: RFLP fragments size (bp)**
INSR/ NsiI	rs2059806	Exon 8 (A/G)	5′-CGGTCTTGTAAGGGTAACTG-3′	93°C, 45 s,56°C	NsiI, 37°C	NsiI, 37°C	Allele G: 324 Allele A: 239 + 85
			5′-GAATTCACATTCCCAAGACA-3′	30 s, 72°C 45 s			
INSR/PmlI	(rs1799817)	Exon 17 (T/C)	5′-CCAAGGATGCTGTGTAGATAAG-3′	93°C, 45 s,56°C	317	PmlI, 37°C	Allele T: 317 Allele C: 274 + 43
			5′-TCAGGAAAGCCAGCCCATGTC-3′	30 s, 72°C 45 s			
ADIPOQ/SmaI	(rs2241766)	Exon 2 (T/G)	5′-GAAGTAGACTCTGCTGAGATG G-3′	93°C, 45 s,56°C	372	SmaI, 30°C	Allele T: 372 Allele G: 216 +156
			5′-TATCAGTGTAGGAGGTCTGTGATG-3′	30 s, 72°C 45 s			
ADIPOQ/BsmI	(rs1501299)	Intron 2 (C/A)	5′-GGCCTCTTTCATCACAGACC-3′	93°C, 45 s,58°C	196	BsmI, 37°C	Allele A: 196 Allele C: 146 + 50
			5′-AGATGCAGCAAAGCCAAAGT-3′	30 s, 72°C 45 s			

* Single nucleotide polymorphisms.

**Table II:** Characteristics of study participants

**Variables**		**PCOS** **(n=186)**	**Controls** **(n=156)**	**p-value**
Age (years)		26.6 ± 5.6*	30.8 ± 5.6*	<0.001**
% subjects with history of primary infertility		28.7%	8.9%	<0.001®
% subjects with family history of hirsutism		47.5%	14.0%	<0.001®
Blood pressure				
	Systolic (mmHg)	106 ± 12.3*	108 ± 11.8*	0.14**
	Diastolic (mmHg)	68.5 ± 10.1*	67.7 ± 9.1*	0.44**
Body mass index (Kg/m^2^)		26.8 ± 6.4*	25.5 ± 4.4*	0.03**
Waist circumferences (m)		0.87 ± 0.13*	0.85 ± 0.09*	0.28**
Hip circumferences (m)		1.03 ± 0.1*	1.0 ± 0.10*	0.01**
Waist to hip ratio		0.8 ± 0.07*	0.9 ± 0.6*	0.22**
Total testosterone		0.7 (0.4-1.2)^●^	0.4 (0.3-0.6)	0.04^●●^
Androstendion		2.4 (2-2.8)^●^	1.7 (1.1-2.2)	0.15^●●^
Dehydroepiandrosterone sulfate		115 (91-241)^●^	120 (79-160)	0.38^●●^
LH/FSH		1.1 (0.7-1.8)^●^	1.0 (0.6-1.7)	0.44^●●^
^α^HOMA-IR		2.3 (1.6-3.7)^●^	2.0 (1.3-2.8)	0.09^●●^

Hormonal tests were only assessed for 40 women of control group.

*Mean ± SD                    ^●^ Median (interquartile range)                    **T-Test                      ^●● ^Mann-Whitney

**® **Chi-square                    ^α^Homeostasis Model of Assessment - Insulin Resistance

**Table III:** Genotype and allele frequencies of studied SNPs in the case and control groups

**Genotype frequency (%)**				**PCOS** ^ a^ **(n= 186)**	**Controls** **(n= 156)**
Adiponectin					
	SmaI				
			GG	2 (1.1)	4 (2.6)
			TT	142 (76.3)	106 (67.9)
			TG	42 (22.6)	46 (29.5)
	BsmaI				
			CC	92 (49.5)	77 (49.4)
			AA	18 (9.7)	8 (5.1)
			CA	76 (40.9)	71 (45.5)
Insulin					
	PmlI				
			TT	114 (61.3)	95 (60.9)
			CC	15 (8.1)	7 (4.5)
			TC	57 (30.6)	54 (34.6)
		NsiI			
			GG	11 (5.9)	18 (11.5)
			AA	99 (53.2)	80 (51.3)
			AG	76 (40.9)	58 (37.2)
Allele frequency (%)					
	SmaI				
			T	144 (77.4)	110 (70.5)
			G	42 (22.6)	46 (29.5)
	BsmaI				
			A	92 (49.5)	77 (49.4)
			C	94 (50.5)	79 (50.6)
	PmlI				
			T	114 (61.3)	95 (60.9)
			C	72 (38.7)	61 (39.1)
	NsiI				
			A	110 (59.1)	98 (62.8)
			G	76 (40.9)	58 (37.2)

^a ^Chi-square have shown that there were no statistical significant differences in allele and genotype distributions between these two groups.

**Table IV:** The relationship between haplotype of Adiponectin and insulin two polymorphisms in the two study groups

		**PCOS**										**Controls**								
		**TA (n=63)**		**TC (n=81)**		**GA (n=29)**		**GC (n=13)**		**p-value**		**TA (n=48)**			**TC (n=62)**		**GA (n=29)**		**GC (n=17)**	**p-value**
**Haplotype adiponectin**																				
Body mass index (kg/m ^2^)	26.6±4. 9		26.6±7.31		27.6±6.16		26.7±7.91		0.91		25.00±4.03		26.00±4.65			25.7±4.95		24.3±3.71		0.46
PCO- severity score	9.13±3		9.15±2.88		29.0±2.71		9.23±1.69		0.90		3.48±1.20		3.26±0.60			3.24±0.69		3.29±0.59		0.53
Systolic (mmHg)	107±11.6		9.15±2.88		109±13.0		108±11.1		0.14		108±11		109±13.3			106±10.7		108±10.1		0.80
Diastolic (mmHg)	69.2±8.7		67.1±10.6		69.1±10.2		73.5±10.9		0.11		67.6±8. 9		68.5±9.2			65.5±9.1		68.8±9.9		0.50
Waist Circumferences (m)	0.87±0.11		0.86±0.13		0.89±0.14		0.9 ±0.17		0.52		0.85±0.09		0.88±0.09			0.85±0.1		0.8. ±0.07		0.03
Hip Circumferences (m)	1.04±0.08		1.02±0.10		1.05±0.12		1.05±0.14		0.64		1.00±0.08		1.01±0.14			1.01±0.09		0.99±0.07		0.91
WHR^®^	0.8±0.1		0.8±0.07		0.8±0.06		0.8±0.06		0.70		0.8±0.05		0.1±0.9			0.83±0.05		0.8±0.04		0.56
HOM-IR^α^	1.2(0.7-1.9)		1.1(0.7-1.8)		1.2(0.5-2)		0.8(0.5-2)		0.37^●^		-		-			-		-		-
LH/FSH	2.4(1.2-3.4)		2.1(1.7-3.5)		3.7(1.5-8)		1.6(0.7-2)		0.95^●^		-		-			-		-		-
	**PCOS**										**Controls**									
	**CA (n=62)**		**CG (n=52)**		**TA (n=48)**		**CG (n=24)**		**p-value**		**CA (n=61)**		**CG (n=34)**			**TA (n=37)**		**CG (n=24)**		**p-value**
**Haplotype Insulin**																				
Body mass index (kg/m ^2^)	27.1±6.7		26.4±7.2		26.9±5.4		26.3±6.1		0.90		25.4±4.2			24.2±3.4		26.4±5.1		25.9±4.9		0.20
PCO- severity score	9.5±2.9		9.13±2.9		8.64±2.5		9.58±2.9		0.44		3.3±0.7			3.2±0.4		3.3±1.1		3.4±1.02		0.66
Systolic (mmHg)	105±11.7		106±11.8		107±12.9		105±13.9		0.60		106±11.9			109±12.5		110±11.7		108±10.7		0.31
Diastolic (mmHg)	67.1±9.9		68.5±9.1		70.9±10.9		67.6±1		0.23		66.2±8.9			67.1±9.06		69.7±8.9		69.6±9.7		0.20
Waist (m	0.87±0.14		0.86±0.12		0. 9±0.13		0.85±0.12		0.62		0.86±0.09			0.83±0.08		0.87±0.11		0.87±0.09		0.31
Hip (m)	1.05±0.12		1.02±0.9		1.04±0.10		1.02±0.11		0.60		1.01±0.08			0.99±0.05		1.01±0.08		0.99±0.22		0.74
WHR^®^	0.8±0.1		0.84±0.1		0.85±0.1		0.8±0.07		0.47		0.85±0.06			0.83±0.05		0.85±0.06		1.2±1.6		0.11
HOM-IR^α^	2.2(1.7-5)		2.4(1.5-3.1)		2.7(2-3.6)		2.2(0.7-5.5)		0. 83^●^		-			-		-		-		-
LH/FSH	0.9(0.5-1.5)		1.3(0.8-2.3)		1.1(0.5-1.7)		1.8(0.8-1.6)		0.30^●^		-			-		-		-		-

One-way ANOVA          ^●^Mann-Whitney          ^α^Homeostasis Model of Assessment - Insulin Resistance,          ® Waist to hip ratio

Chi-square have shown that there were no statistical significant differences in haplotype distributions between these two groups.

## Discussion

In the present study we assessed whether ADIPOQ or INSR genes polymorphisms are associated with the risk for PCOS among Iranian women and found no significant difference in these two genes allele and genotype frequencies between the case and control groups. 

It is assumed that perhaps ADIPOQ and INSR polymorphisms could be used for the stratification of women at risk of PCOS (19, 24). Contrary to this assumption, we did not find any association between ADIPOQ or INSR genes polymorphisms and PCOS among a sample of Iranian women. Our results are in agreement with those of Escobar-Morreale *et al*, Xita *et al* and Urbanek *et al*, however while Escobar-Morreale *et al* showed no association between resistin -420 C>G polymorphism with PCOS, they found an association between ADIPOQ 45 T>G polymorphism and PCOS (18, 25, 26). 

The 45 G>T polymorphism has been reported to be associated with PCOS among German and Caucasian women (27, 28), while this was not observed in other populations (14, 20, 28). Several studies suggest that insulin resistance in PCOS patients is the result of a defect in INSR signaling (29, 30). There are studies however with various results on the association between insulin and INSR genes polymorphisms with PCOS. An association between polymorphism C/T in the exon 17 INSR gene with PCOS is reported by several studies but other investigations found no similar relation (7, 16, 21, 24, 31). 

The results of the current study showed that the TT and AA genotypes and T and A alleles of PmlI and Nsil polymorphisms of INSR gene are most commonly observed among both PCOS cases and controls, respectively. Furthermore, our results demonstrated that the TT genotype and the T allele of SmaI polymorphism of ADIPOQ gene were the most frequent observations in both case and control groups. 

Nevertheless, our results suggest that two known polymorphisms of ADIPOQ gene (SmaI and BsmaI) and insulin gene (PmlI and NsiI) have similar distributions of the allelic variants in the PCOS and normal group. Recent data reported that C/T polymorphism in the exon 17 of INSR gene is correlated with PCOS in different populations (7, 16, 24). Although some studies reported a relationship between T/C polymorphism of exon 17 of INSR gene and decrease in insulin sensitivity, other investigations found no similar association (7, 21, 31). 

Our data showed that the score for the severity of PCOS among women with the GG genotype were lower than those with TT or TG; while the differences were not statistically significant, possibly due to lack of enough sample size. In addition, we found an association between central obesity and the ADIPOQ haplotype; waist circumstance of women with TC haplotype in the control group was significantly higher in compare with case group. In agreement with our results, Xita *et al*, reported an association between ADIPOQ polymorphisms with obesity in PCOS women; obesity was associated with ADIPOQ gene polymorphism at +276 (18). 

Menzaghi *et al*, found a significant association between the ADIPOQ polymorphism and waist circumference, blood pressure and insulin level (32). Factors contributing to the different results of various studies could be attributable to different criteria for identification of PCOS women, various methods for recruitment of study subjects, the heterogeneity of PCOS subjects, using various methods for genetic analysis and racial differences of study participants. However, our results were subject to two limitations. First, having low sample size that precludes drawing strong conclusions. 

In the present study diagnosis of normo-ovulatory women was made based on having regular and predictable menstruation, without history of infertility, while it has been shown that 3.7% of eumenorrheic, non-hirsute women, have oligo-ovulatory cycles diagnosed by serum concentration of progesterone (33). Second limitation was lacking of measurements of biomarkers such as adiponectin or using the standard method for identification of insulin resistance. The case-control design of our study could potentially bias our results. 

## Conclusion

In conclusion, in this case-control study, we found no significant association between the INSR and adiponectin gene polymorphisms and PCOS risk. Further studies with larger sample sizes are warranted to confirm these findings.
